# Design of Experiments for the Synthesis of MMT/TiO_2_/Ag Composites: Integrating Adsorption and Photocatalysis under Artificial UV and Natural Sunlight for Ethylene Degradation

**DOI:** 10.1021/acsomega.6c01808

**Published:** 2026-06-30

**Authors:** Yuri B. Fávaro, Michel Z. Fidelis, Giane G. Lenzi, Artur J. Motheo, Marcos David Ferreira, Henriette M. C. de Azeredo

**Affiliations:** † Graduate Program in Chemical Engineering (PPGEQ), Federal University of São Carlos (UFSCAR), São Carlos, São Paulo 13565-905, Brazil; ‡ São Carlos Institute of Chemistry, University of São Paulo (USP), São Carlos, São Paulo 13566-590, Brazil; § Department of Chemical Engineering, Federal Technological University of Paraná (UTFPR), Ponta Grossa, Paraná 84017-220, Brazil; ∥ Embrapa Instrumentation, São Carlos, São Paulo 13560-970, Brazil

## Abstract

Postharvest deterioration caused by ethylene (C_2_H_4_) is a major challenge for global food security, leading to significant losses of perishable products like bananas. In this study, we present the synthesis and optimization of a multicomponent photocatalyst based on montmorillonite (MMT), titanium dioxide (TiO_2_), and silver (Ag), designed for efficient ethylene remediation. The catalysts were prepared via wet impregnation and characterized using FE-SEM, EDS, and UV–vis reflectance spectroscopy, which confirmed the successful integration of components and a homogeneous elemental distribution. The bandgap energies were determined to be in the range of 3.02 to 4.10 eV, with MMT-rich samples showing values around 3.12 eV. Through a 2^3^ factorial design, the performance was evaluated under three distinct scenarios: adsorption, artificial UV photocatalysis, and natural sunlight-driven photocatalysis. Sample Amt 09 (1.5% TiO_2_ and 1% Ag) achieved an ethylene removal of over 92% in the artificial UV photocatalysis scenario. Sample Amt 10 (5% MMT, 1.5% TiO_2_, and 1% Ag) stood out with high performance across all scenarios, suggesting synergistic effects between adsorption and catalytic degradation. Furthermore, an optimized formulation (5% MMT and 2.73% TiO_2_) achieved 65% ethylene removal under UV light and 47% under natural sunlight. These findings highlight the potential of MMT/TiO_2_/Ag composites as sustainable, light-driven solutions for postharvest management, offering a scalable platform to extend the shelf life of fresh produce and reduce global food waste.

## Graphical abstract

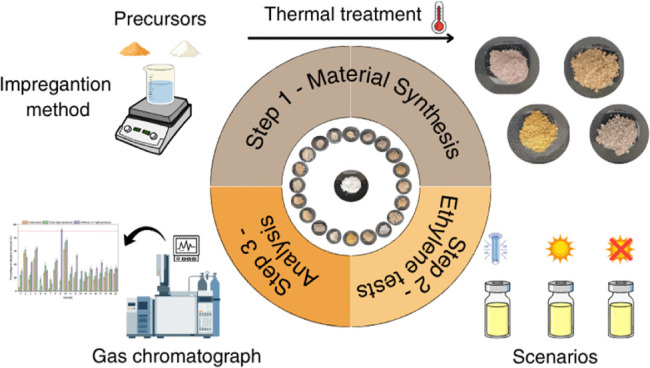

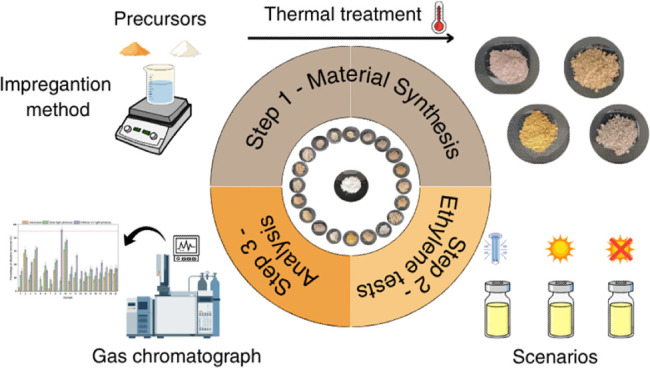

## 1 Introduction

The preservation of perishable agricultural products remains one of the most significant challenges for global food security and sustainability. It is estimated that approximately one-third of all food produced for human consumption is lost or wasted, with postharvest deterioration being one of the primary contributing factors.^1^ Among the various factors influencing the shelf life of fresh produce, ethylene (C_2_H_4_) plays a predominant role as a gaseous phytohormone that coordinates the ripening, softening, and senescence of climacteric fruits. This occurs through irreversible biochemical changes, such as the conversion of complex polysaccharides into simple sugars, chlorophyll degradation, and the synthesis of volatile aromatic compounds.^2,3^ For instance, bananas are particularly sensitive to ethylene, exhibiting a climacteric peak with ethylene production rates ranging between 0.1 and 50 μL kg^–1^ h^–1^, depending on the cultivar and environmental conditions.^4–6^

Traditional strategies to mitigate ethylene-induced deterioration have focused primarily on physical and chemical interventions. Physical methods, such as low-temperature storage and controlled atmosphere (CA) systems, aim to reduce the fruit’s metabolic rate, suppressing both respiration and ethylene biosynthesis. Nevertheless, these systems require substantial energy consumption and specialized infrastructure.^7,8^ Chemical methods, notably the use of potassium permanganate (KMnO_4_) as an oxidant, are effective and widespread. They face, nonetheless, limitations regarding chemical waste disposal due to their toxicity, particularly concerning water contamination.^9–12^

Advanced oxidation processes (AOPs), specifically photocatalytic oxidation, are emerging as an alternative for ethylene removal in fruits such as bananas,^13–16^ papayas,^17–19^ and strawberries.^14,20,21^ For example, photocatalysis using titanium dioxide (TiO_2_) is considered effective due to its high stability, low toxicity, and potent oxidative capacity.^22^ Under irradiation with light energy exceeding its bandgap, TiO_2_ generates electron–hole pairs (e^–^/h^+^) that react with moisture and oxygen on the catalyst surface to produce highly reactive species, such as hydroxyl radicals (•OH) and superoxide anions $\left(O_{2}^{•-}\right)$ .^22–24^ Yet, the adoption of pure TiO_2_ is hindered by two points: its wide bandgap energy (3.2 eV with absorption at ∼387 nm in the anatase phase), which limits its activity to the ultraviolet (UV) portion of the spectrum,^25,26^ and the rapid recombination rate of photogenerated charge carriers, which decreases the overall quantum efficiency.^27–30^

To bypass these limitations, current research has turned toward the engineering of multicomponent composites that combine semiconductors with noble metals as cocatalysts, and high-surface-area supports. The use of clay minerals, such as montmorillonite (MMT), as support matrices is being studied. MMT is a 2:1 phyllosilicate characterized by a lamellar structure with high cation exchange capacity and high specific surface área,^31,32^ in addition to providing improvements in barrier, mechanical, and adhesive properties.^33–36^ Silver (Ag) nanoparticles as cocatalysts have demonstrated efficacy in electron trapping, preventing recombination,^37–41^ and acting as effective inhibitors of ethylene action.^42–44^

Recent advances in photocatalysis have focused on the development of ternary nanocomposites to overcome the limitations of pure TiO_2_. For instance, the integration of p-type semiconductors like CuO and La_2_O_3_ into TiO_2_ matrices has been shown to significantly reduce bandgap energy and enhance charge carrier separation through the formation of p-n heterojunctions or Z-scheme systems. Studies by Boudraa et al. demonstrated that such modifications can extend the photocatalytic activity into the visible light spectrum, achieving high degradation efficiencies for organic pollutants like malachite green and brilliant green. While these strategies have shown unprecedented efficacy in aqueous media, their application for the remediation of gaseous phytohormones like ethylene under high-concentration scenarios remains a challenge.^45,46^

To address this complexity, the application of Design of Experiments (DoE) and Response Surface Methodology (RSM) is essential. Statistical modeling allows for the simultaneous evaluation of multiple variables, identifying not only the individual effects of MMT, TiO_2_, and Ag concentrations but also their mutual interactions. This approach ensures that the resulting material is not merely a combination of its parts, but a finely tuned system optimized for maximum ethylene degradation efficiency.

This study distinguishes itself by optimizing an MMT/TiO_2_/Ag composite specifically designed to integrate high adsorption capacity with photocatalytic degradation, targeting practical postharvest environments where sunlight or artificial UV may be inconsistently available. Using a 2^3^ factorial design with replicates at the center point, we investigated the influence of the components on the physicochemical properties of the catalyst, including bandgap energy, surface morphology, and specific surface area. The performance of the synthesized materials was evaluated across three distinct reaction scenarios: adsorption in the absence of light, photocatalysis under artificial UV light, and photocatalysis under natural sunlight. Furthermore, for subsequent stages, the optimized catalyst can be incorporated into a natural polymeric matrix to act as a functional coating for bananas or integrated into smart packaging and applied to films as adsorbents, bridging fundamental materials science with practical postharvest management solutions. This work contributes to the development of sustainable technologies to reduce global food waste and increase the commercial shelf life of fresh produce.

## 2 Materials and Methods

### 2.1 Chemicals

Montmorillonite (MMT) (K10 Montmorillonite, Sigma-Aldrich, Lot: STBH6207), Titanium Dioxide (TiO_2_) (anatase, 99.8%, Sigma-Aldrich, Lot: MKCG3985), Silver Nitrate (AgNO_3_, Plat-Lab, Lot: 1888/22), Deionized (DI) water (18 MΩ) and Ethylene (White Martins, 99.5% purity).

### 2.2 Design of Experiments (DoE)

The applied Design of experiments (DoE) was a 2^3^ factorial design with six center point replicates (Table 1). The design aimed to optimize the concentrations of Montmorillonite (MMT), Titanium Dioxide (TiO_2_), and silver (provided via the AgNO_3_ precursor) during the catalyst synthesis, with the response being the residual ethylene concentration in the reaction.

**Table 1:** Design of Experiments for Catalyst Synthesis

Sample	MMT (%)	TiO_2_ (%)	Ag (%)
CTR	0.00	0.00	0.00
Amt 1	1.01	0.61	0.41
Amt 2	3.99	0.61	0.41
Amt 3	1.01	2.39	0.41
Amt 4	3.99	2.39	0.41
Amt 5	1.01	0.61	1.59
Amt 6	3.99	0.61	1.59
Amt 7	1.01	2.39	1.59
Amt 8	3.99	2.39	1.59
Amt 9	0.00	1.50	1.00
Amt 10	5.00	1.50	1.00
Amt 11	2.50	0.00	1.00
Amt 12	2.50	3.00	1.00
Amt 13	2.50	1.50	0.00
Amt 14	2.50	1.50	2.00
Amt 15	2.50	1.50	1.00
Amt 16	2.50	1.50	1.00
Amt 17	2.50	1.50	1.00
Amt 18	2.50	1.50	1.00
Amt 19	2.50	1.50	1.00
Amt 20	2.50	1.50	1.00

For the application step of the tests, ethylene (White Martins, 99.5% purity) and hermetically sealed 25 mL glass vials were used, equipped with a pressure cap and a silicone septum (Purion lab products, lot number: 306077302, part number: C2203, China). The CTR sample, containing no catalytic compounds, served as the experimental blank. In this control, only ethylene gas was introduced into the vial, ensuring that any degradation observed in the other samples could be attributed solely to the catalytic processes.

### 2.3 Catalyst Synthesis

The catalysts were synthesized using the classic wet impregnation method, with adaptations based on the methodology proposed by Lenzi et al.^47^ Initially, the materials MMT, TiO_2_ and AgNO_3_ (whose concentrations were previously determined by the experimental design shown in Table 1) were added to 20 mL of deionized water and magnetically stirred at 70 °C until a paste was formed. Subsequently, the resulting product was dried for 24 h in a forced air circulation oven at 100 °C (Solab, Brazil, SL-102). A portion of this material was then subjected to thermal treatment.

The synthesized materials underwent thermal treatment using a two-stage thermal ramp with a heating rate of 5 °C^1–^, followed by a holding time of 180 min at the final temperature in a muffle furnace (EDG, 1800, Brazil). Since anatase was employed as the starting precursor, this temperature was selected to maintain the crystalline stability of the TiO_2_ phase while ensuring the removal of moisture and residual impurities. Also, to promote the thermal decomposition of AgNO_3_ and the subsequent reduction of silver species. At this temperature, a controlled decomposition occurs, favoring the formation of highly dispersed silver nanoparticles and clusters on the MMT/TiO_2_ surface, while avoiding excessive sintering that could reduce the active surface area.^48,49^

### 2.4 Characterization

All material characterizations were carried out at Embrapa Instrumentation, São Carlos-SP, Brazil. The reflectance values for the samples were obtained using a spectrophotometer (Shimadzu, UV-2600, Japan) with a reading range between 185 and 900 nm. The integrated ISR-2600 sphere, which is coupled to the equipment, allows for readings of wavelengths between 220 and 1400 nm and enables measurements of transmittance and diffuse reflectance in solid samples. The signals obtained from the catalysts were processed using the UVProbe 2.7 software, which also allows for the conversion of reflectance data into absorbance via the Kubelka–Munk transformation. The surface of the catalysts was analyzed using a Scanning Electron Microscope (SEM) (Jeol, JMS 6510, Japan) coupled with a Thermo System Seven EDS microanalysis system and the NSS software. The samples were predried in a desiccator with silica gel, fixed onto metallic supports (stubs) using double-sided carbon tape, and then carbon-coated via sputtering in a sputtering device (Leica, EM SCD050). Micrographs were obtained with an acceleration voltage of 5 kV, a Working Distance (WD) of 10 mm, and a magnification of 2000x. The surface area analysis of the materials was performed using an ASAP 2020 instrument (Micromeritics Instrument Corporation, USA). Approximately 0.22 mg of each sample was added to a sample tube and subjected to a pretreatment step to clean the material surface by applying a vacuum of 10 μmHg. Subsequently, a thermal treatment was performed with a heating ramp of 10 °C.min^–1^ up to 100 °C, where it was maintained overnight to remove moisture and volatile compounds from the solid. After this process, the sample was reweighed and subjected to the analysis using gaseous nitrogen (N_2_) as the adsorbate, at a temperature of 77 K. The specific surface area was determined using the Brunauer–Emmett–Teller (BET) model, considering a relative pressure range (P/P_0_) between 0.05 and 0.30.

### 2.5 *In Vitro* Tests

The *in vitro* tests were conducted at the São Carlos Institute of Chemistry, University of São Paulo, São Carlos-SP, Brazil, under three distinct reactional conditions: (i) photocatalysis under artificial UV light, (ii) adsorption, and (iii) photocatalysis under natural (solar) light. Approximately 60 μL of ethylene, corresponding to a concentration near 5000 ppm, was injected into 25 mL hermetically sealed glass vials equipped with a pressure cap and a silicone septum (Purion lab products, lot number: 306077302, part number: C2203, China) and maintained for 120 min for each reaction. Each flask contained the catalyst at concentrations previously established according to the DoE (Design of experiments). The selection of this ethylene quantity aimed to minimize the error associated with catalyst weighing, given that the masses utilized were extremely small. Furthermore, it is highlighted that Yi et al. reported an ethylene production rate in bananas of 1.7 μL kg^–1^ h^–1^, a value used as a reference to estimate the experimental conditions.^50^ The reaction conditions are *Photocatalysis Artificial UV Light:* The photocatalytic reaction under artificial UV light was conducted in a closed chamber with air circulation, equipped with a high-pressure mercury vapor lamp (Philips), without its protective bulb. This lamp operated with a fluence rate of 0.113 W cm^–2^ and was positioned approximately 10 cm away from the flasks containing the catalyst and ethylene; *Adsorption:* For the adsorption system, the flasks were wrapped in aluminum foil and maintained in a closed environment, ensuring no incidence of natural or artificial light; *Photocatalysis Natural Solar Light:* In the third system, intended for photocatalysis under natural light, the flasks were placed in a location with good solar light incidence.

### 2.6 Gas Chromatographic Analysis

After the reaction time elapsed (120 min), a 1 mL aliquot of the content from each flask was injected into a Gas Chromatograph (GC) (Thermo Scientific, Trace 1310, Italy) at Embrapa Instrumentation, São Carlos-SP, Brazil. The GC was equipped with a split/splitless injector and both a Thermal Conductivity Detector (TCD) and a Flame Ionization Detector (FID) operating in parallel. Signal acquisition and chromatographic analysis were performed using the ChromQuest 5.0 software, supplied with the instrument. Ethylene was identified using the FID detector, exhibiting a retention time of approximately 5 min.

## 3 Results and Discussion

### 3.1 Diffuse Reflectance Spectrophotometry (DRS)

Based on the developed experimental design, a total of 20 catalysts were synthesized: 15 corresponding to different combinations of the studied variables and 5 corresponding to the center point replicates. After the calcination process at 400 °C, a noticeable alteration in the color of the catalysts was observed. This change is related to the variations in the concentrations of montmorillonite, titanium dioxide, and silver, which directly influence the material structure after the thermal treatment. The absorbance spectra of the samples were obtained using diffuse reflectance spectrophotometry and subsequently processed using the Tauc equation given by, (*αhv*)*^n^* = *A*(*hv* – *E_g_*), assuming n = 2. Where α is the absorption coefficient, A is a constant dependent on the optical transition of the material under investigation, *hν* is the photon energy, E_g_ is the band gap energy and n depends on the nature of the optical transition in the semiconductor, assuming the value 2 or 0.5 for indirect or direct optical transitions, respectively.^51^ The resulting band gap energy (E_g_) values are presented in Figure 1.

**Figure 1:**
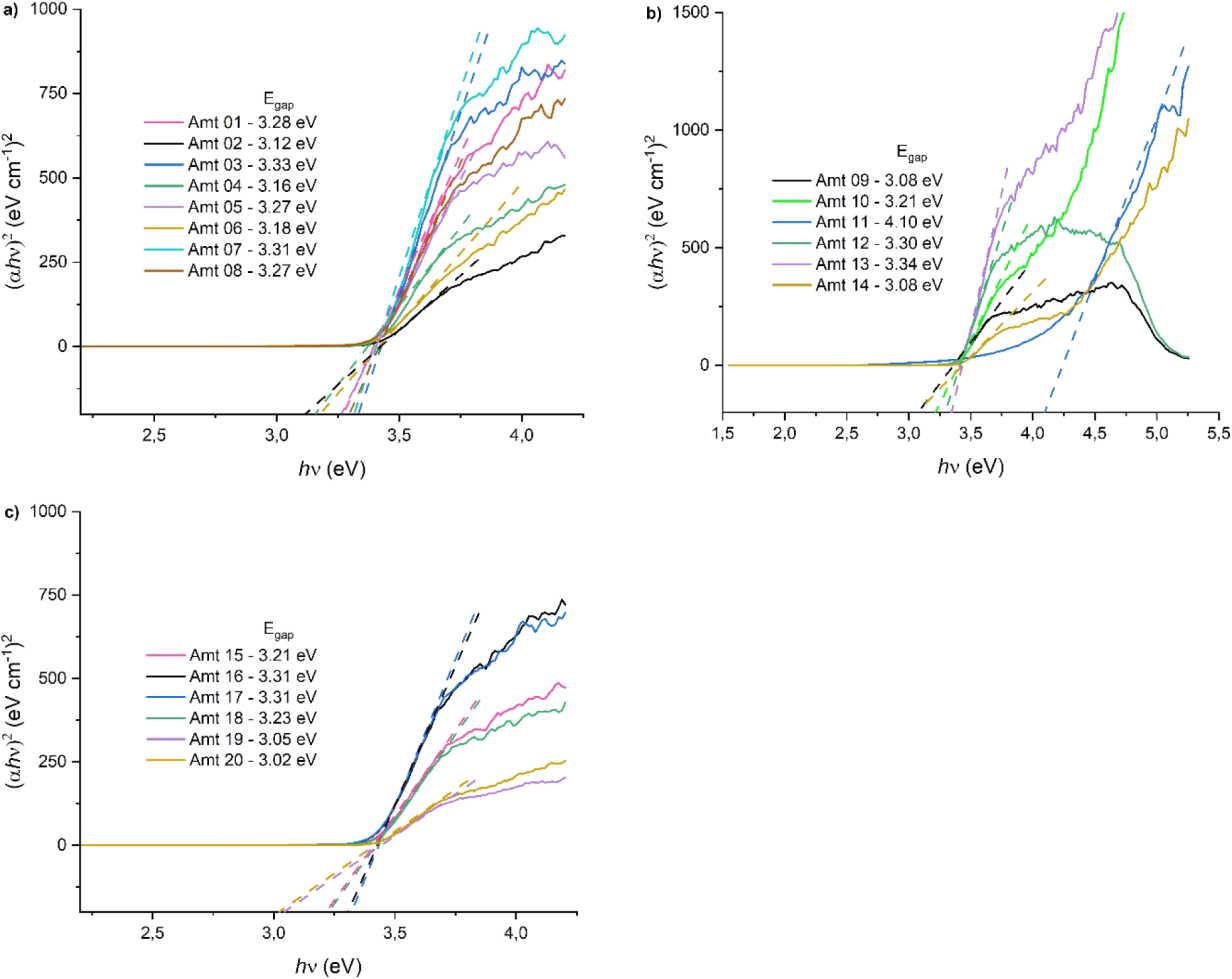
Band gap energy (E_g_) of the catalyst samples of: factorial points (a); central points (b); axial points (c).

The data shown in Figure 1 present the band gap values for samples calcined at 400 °C. For the axial point samples containing a higher MMT concentration in the MMT:TiO_2_ ratio, lower band gap energy values were obtained (3.12–3.27 eV) compared to samples with higher or balanced TiO_2_ concentrations (3.28–3.31 eV). Although the component proportions and preparation methods used in the present study differ, Olad et al. prepared and characterized a nanocomposite containing silver ion exchange in montmorillonite/TiO_2_.^52^ They observed a considerable reduction in band gap energy, from 5.12 eV for pure MMT to approximately 3 eV in the composite, consistent with the findings of Tuo et al., who utilized MMT as a support for TiO_2_ and observed a band gap value of 2.98 eV, representing a reduction of 0.14 eV relative to pure TiO_2_ (3.12 eV).^53^ Similar results were reported by Indress et al., who employed MMT as a support for polyaniline nanostructures.^54^ The chemical interaction promoted by the synthesis led to good dispersion of the MMT within the polymer matrix, resulting in a decrease in the band gap of the original polymer, from 3.71 to 2.88 eV.

### 3.2 Surface Morphology by FE-SEM and EDS

For the surface investigation using FE-SEM and EDS, samples Amt 9, Amt 10, Amt 11, Amt 13, and Amt 21 (both calcined and noncalcined) were selected. The aim of this selection was to provide an overview of the developed experimental design. The corresponding results can be observed in Figure 2.

**Figure 2:**
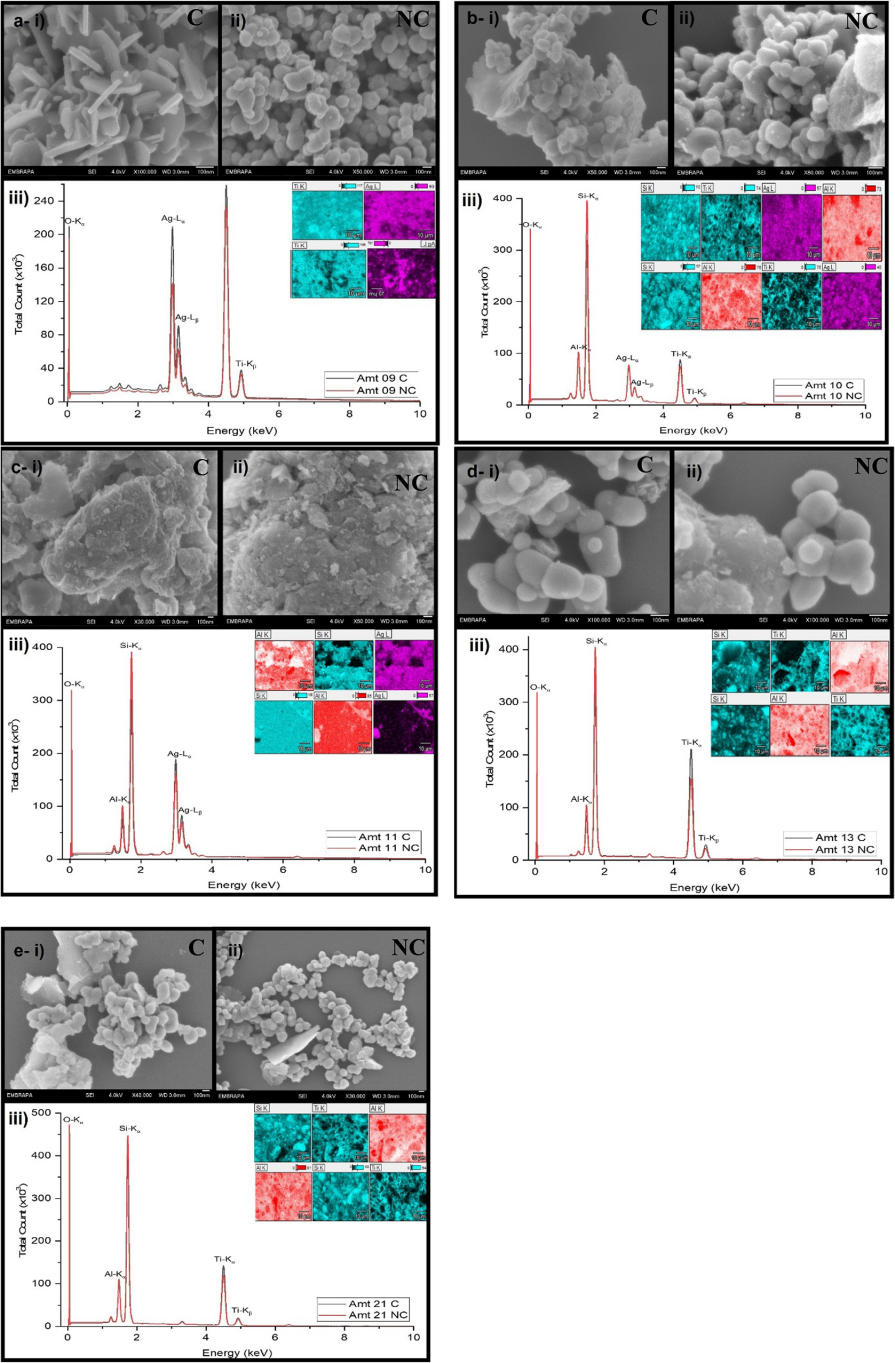
FE-SEM micrographs and EDS elemental mapping of selected composite samples: (a) Amt 09 (1.0% Ag and 1.5% TiO_2_), (b) Amt 10 (5.0% MMT, 1.0% Ag, and 1.5% TiO_2_), (c) Amt 11 (2.5% MMT and 1.0% Ag), (d) Amt 13 (2.5% MMT and 1.5% TiO_2_), and (e) Amt 21 (5.0% MMT and 2.73% TiO_2_).

From the mapping obtained through EDS analysis, a homogeneous dispersion of the elements is observed across the materials, in addition to a similarity in the counts between the calcined and noncalcined samples, as can be analyzed in the graphs. The images generated by FEG-SEM (Field Emission Gun Scanning Electron Microscopy) evidence that the impregnation method provided a good interaction between the materials. In Figure 2a, the noncalcined material containing Ag and TiO2 exhibits a circular structure before the thermal treatment at 400 °C, but it takes on a petal-like shape after calcination. Despite undergoing the same thermal process, the materials containing MMT and TiO2 (Figure 2d), MMT and Ag (Figure 2c) e MMT, Ag and TiO2 (Figure 2b and e) do not undergo this structural alteration. Nevertheless, it is noted that the circular structures are superimposed or connected with the lamellar structures, showing a good interaction of the materials during the synthesis process.

### 3.3 FTIR Spectra

The FTIR spectra obtained are presented in Figure 3. The analyzed samples, calcined at 400 °C, exhibit characteristic bands that confirm the presence of the main components: montmorillonite (MMT), titanium dioxide (TiO_2_), and silver (Ag). Furthermore, the spectra provide evidence of associated structural or chemical modifications and reactions involving ethylene due to the different treatments applied. A band is observed at 3622 cm^–1^, which is attributed to the *v*(O–H) stretching vibration of structural hydroxyl groups bonded to aluminum and silicon (Al–OH and Si–OH). These groups are situated between the octahedral and tetrahedral layers of the montmorillonite structure. In the 2355 cm^–1^ region, a band is identified that is associated with both the stretching of OH groups from adsorbed water molecules and the absorption of atmospheric CO_2_, which is often present during FTIR analysis. The band at 1622 cm^–1^ is assigned to the δ­(H–O–H) bending vibration of interlayer water, indicating the presence of adsorbed water molecules within the interlayer space of the MMT lamellar structure.^55–57^

**Figure 3:**
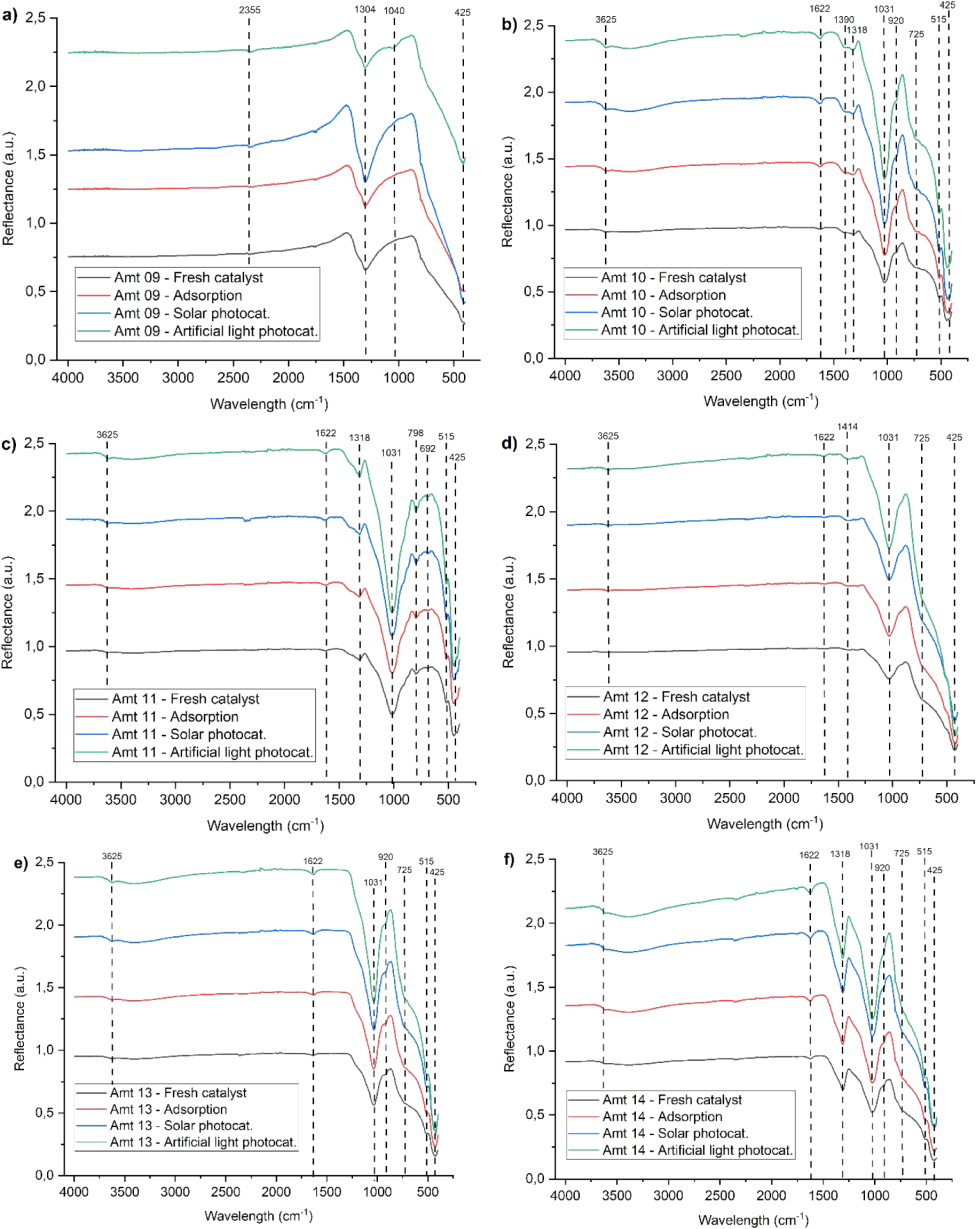
Graphs generated from the FTIR spectra under different scenarios: untreated (as prepared), adsorption, photocatalysis with natural light (solar), and photocatalysis with artificial (UV) light. Sample Amt 09 (a), Sample Amt 10 (b), Sample Amt 11 (c), Sample Amt 12 (d), Sample Amt 13 (e), and Sample Amt 14 (f).

The band at 1390 cm^–1^ may be related to the bond between TiO_2_ and Ag, indicating the successful deposition of silver on the semiconductor surface. In contrast, the signals at 1318 cm^–1^ and 1304 cm^–1^ belong to the typical region for the asymmetric stretching of the nitrate ion (NO_3_^–^). This suggests the presence of residues from the AgNO_3_ precursor that were not completely decomposed during the impregnation process.^58^

The intense band observed in the 1031–1040 cm^–1^ region is characteristic of the asymmetric stretching vibration of the Si–O–Si and Si–O–Al groups, which are typical of the tetrahedral network of montmorillonite. In this same context, additional bands, such as the one observed at 920 cm^–1^ (or 913 cm^–1^), are attributed to the Al–Al–OH deformation vibration, which is related to the octahedral structure of the clay mineral. The peak at 798 cm^–1^ is associated with the Al–O–Si vibration group, while the band at 725 cm^–1^ corresponds to bending modes attributed to the Ti–O–Ti bond.^55,59^ The signal at 692 cm^–1^ may be related to the silicate ring vibrations present in the MMT structure. In the lower wavenumber regions, bands are prominent, notably the one at 515 cm^–1^, which is attributed to the δ­(Al–O–Si) deformation, and the band at 425 cm^–1^. The latter may represent Ti–O–Ti vibrations (anatase/rutile), Ti–O–Si or Si–O in hybrid materials, in addition to possible structural changes resulting from the presence of Ag^+^ ions. Furthermore, the band at 418 cm^–1^ is characteristic of the Ti–O vibration, thus confirming the presence of titanium dioxide.^59–62^

Comparison among the different treatments reveals significant alterations in the spectral profiles. Overall, the photocatalytic treatment under artificial UV light promoted the most substantial changes, evidenced by the increase in intensity of the bands related to Ti–O, Si–O, and residual nitrate. This behavior can be associated with the surface activation of the material, an increase in contact area, or the formation of new functional groups following exposure to radiation and reaction with adsorbed molecules. The application of the photocatalysis reaction under natural (solar) light of the material with ethylene also resulted in the intensification of certain bands, albeit to a less pronounced extent, suggesting moderate surface interactions with the compounds present in the medium. In contrast, the adsorption and synthesis process caused specific, localized changes, notably the increase in the NO_3_^–^ band, which indicates the retention of nitrogenous species in active surface regions. This suggests that the thermal decomposition of the AgNO_3_ precursor at 400 °C was not exhaustive, leading to a coexistence of ionic silver (Ag^+^) and metallic silver (Ag^0^). Conversely, the untreated samples exhibited milder and less complex bands, reflecting the original structure of the materials prior to any chemical or physical modification.

The crystalline structure of the MMT/TiO_2_/Ag composites was further investigated by XRD (Figure S1). The diffractograms for representative samples (Samples 10 and 30) primarily exhibit the characteristic peaks of anatase TiO_2_ and the silicate phases associated with montmorillonite, confirming that the structural integrity of the supports was maintained after the impregnation and calcination processes. No diffraction peaks corresponding to metallic silver (Ag^0^) or silver oxides (Ag_2_O) were detected. This absence is attributed to the low silver loading and its high state of dispersion as small nanocrystallites or amorphous clusters across the composite surface, which fall below the detection limit of the technique. Such high dispersion is consistent with the broad NO_3_^–^ bands observed in FTIR, suggesting that the silver species are effectively distributed in the active regions of the catalyst.

### 3.4 Surface Area Analysis

Surface area (S_g_), pore volume (PV), mean particle diameter (D_m_) and number of pores (N_porous_) are presented in Table 2. The values were obtained through N_2_ adsorption–desorption isotherm analysis and collected using the ASAP 2020 software.

**Table 2:** BET Surface Area, Pore Volume, Mean Particle Diameter, and Number of Pores of Composite Samples

Sample	S_g_ (m^2^ g^–1^)	PV (cm^3^ g^–1^)	D_m_ (Å)	N_porous_ (×10^15^)
Amt 09	2.8212	0.0166	257.5772	0.0019
Amt 10	72.8303	0.1525	83.7787	0.4953
Amt 11	26.3266	0.1041	158.2394	0.0502
Amt 13	131.7996	0.1954	59.3050	1.7892
Amt 21	145.2868	0.2153	59.2734	1.9745
MMT	227.5237	0.3381	59.4466	3.0737

The samples presented in this work, all calcined at 400 °C except for the MMT sample, were selected to provide an overview of the different materials studied. The following samples were considered: Sample Amt 9, containing TiO_2_ and Ag; Sample Amt 10, which combines the three components TiO_2_, Ag and MMT) and exhibited the best performance in the natural (solar) light photocatalysis and adsorption scenarios; Sample 11, without the presence of TiO_2_; Sample Amt 13, composed only of MMT and TiO_2_; Sample Amt 21, corresponding to the optimal condition suggested by the software; and finally, the pure MMT sample, used as a comparison material.

Given these results, it is observed that Samples Amt 13 and Amt 21 exhibit the highest surface areas, in addition to mean particle diameters similar to that of the MMT sample. Nevertheless, these samples undergo a reduction in surface area compared to their precursors. According to Kun et al., this decrease may be related to the synthesis reactions, which can cause structural changes in the montmorillonite, resulting in the shift of the original reflection to lower angles.^63^

Sample Amt 9 exhibited the lowest surface area, as well as the lowest number and volume of pores among the samples analyzed. According to Lenzi et al., this behavior suggests that the impregnation methodology may promote the partial filling of free TiO_2_ pores by silver, thereby reducing the available surface area.^47^ The presence of silver, in some systems, can exert a negative effect, acting to obstruct the pores of the support materials. This shadowing effect may be due to the high silver loading in the material, which can obstruct light penetration and lead to the metallization of the system.^64^ This effect may have also influenced the parameters of samples Amt 10 and Amt 11. Consonantly, Mishra et al. reported an approximately 30% reduction in surface area when incorporating 3 wt % of silver nanoparticles into TiO_2_/clay composites.^65^

### 3.5 *In Vitro* Test Results

The *in vitro* tests, following the experimental design, were carried out in three scenarios: natural light, adsorption, and photocatalysis. The experimental responses for the percentage of ethylene removed are presented in in mean ± SD for each scenario Figure 4.

**Figure 4:**
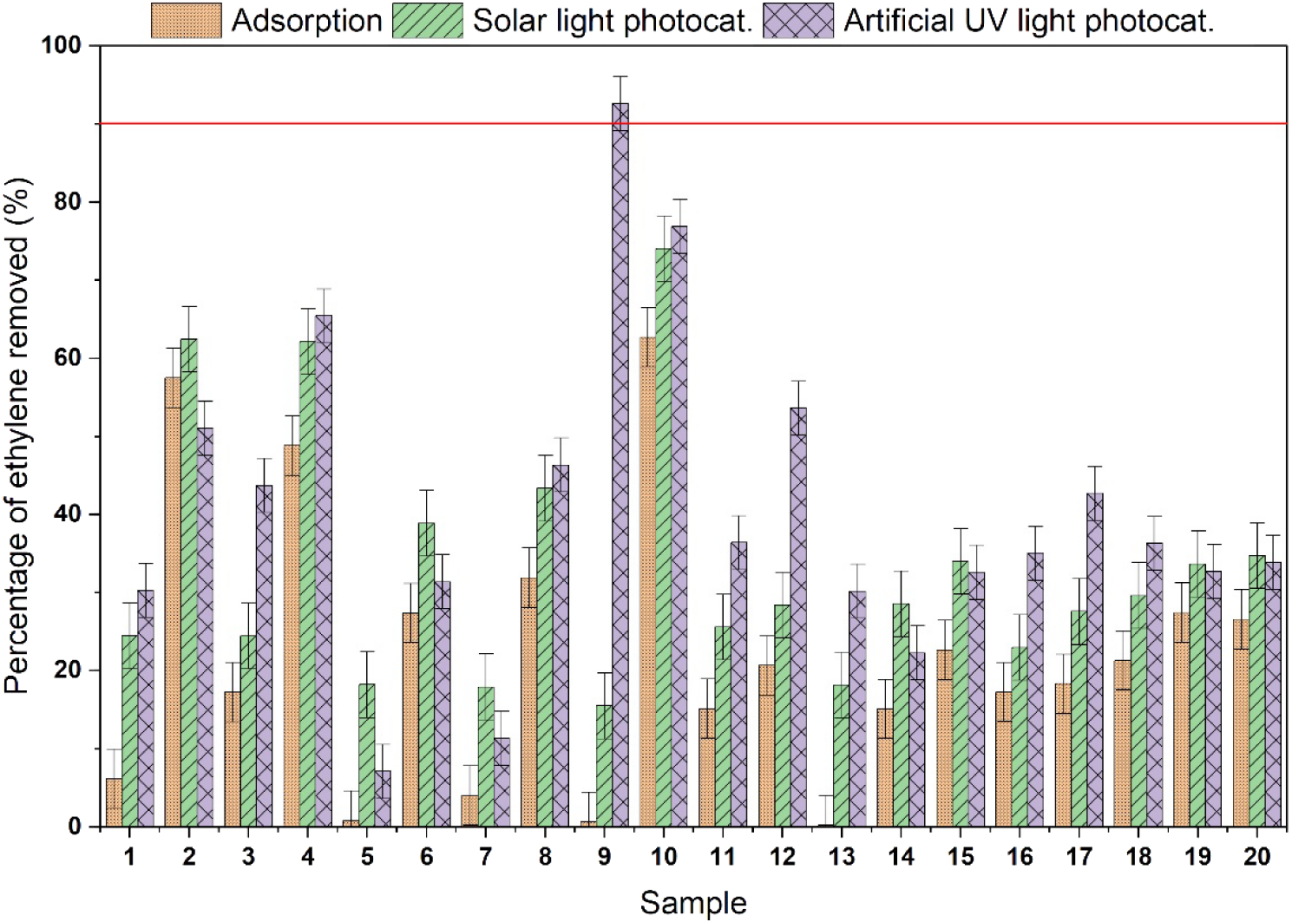
Ethylene removal performance (%) of composite samples in different scenarios: natural solar light photocatalysis, adsorption, and artificial UV light photocatalysis.

Sample Amt 10, which has a concentration of 5% MMT, 1.5% TiO_2_ and 1% Ag, exhibited excellent performance in the natural (solar) light photocatalysis, artificial UV light photocatalysis, and adsorption scenarios. This result can be explained by the higher surface area, as well as the elevated pore volume and number of pores, characteristics that favor adsorption and enhance the photocatalytic action. Sample Amt 9 is also highlighted, as it achieved over 92% ethylene removal under artificial UV light photocatalysis, despite having shown low performance under natural (solar) light photocatalysis and practically zero efficiency in the adsorption scenario. This behavior was expected, given that TiO_2_ is not considered a good adsorbent, requiring UV irradiation for activation, as cited by Linsebigler et al. and Suh et al.^22,66^

It is observed that most of the samples that exhibit higher ethylene removal when subjected to photocatalysis under artificial UV light possess TiO_2_ in the catalyst composition. TiO_2_ is an excellent photocatalyst and can be combined with various materials, such as silver (Ag), to enhance ethylene removal. The use of silver aimed to shift the absorption energy of TiO_2_ into the visible light spectrum and delay the recombination of electron–hole pairs by capturing electrons transferred from the TiO_2_ conduction band and transferring them to the available O_2_.^67^ Furthermore, the Localized Surface Plasmon Resonance (LSPR) effect allows silver to generate hot spots and hot carriers through intense light absorption. Driven by both artificial and solar UV light, this phenomenon significantly accelerates the oxidation of ethylene gas.^68^ For example, Fonseca et al., reported ethylene removal exceeding 60% when applying a gelatin-TiO_2_ coating on papayas.^17^ Siangyai et al., achieved a reduction greater than 85% using zeolite-TiO_2_ based catalysts. Although the photocatalytic tests under artificial UV light yielded satisfactory results, the samples, when evaluated in the natural (solar) light photocatalysis scenario, achieved expressive performances, in some cases even superior to those observed under the artificial UV light photocatalysis condition.^69^

It is highlighted that the samples with higher MMT concentrations (such as Samples Amt 2, Amt 4, Amt 6, Amt 8, and Amt 10) exhibited greater efficiency in ethylene removal in all scenarios when compared to those with lower MMT concentrations. This behavior is attributed to the fact that montmorillonite not only expands the available surface area but also possesses a lamellar structure and surface charge that promote homogeneous dispersion and size control of Ag and TiO_2_ particles, thereby minimizing material agglomeration.^70–72^

It is also observed that when there is a proximity between the concentrations of the constituents, especially when the silver concentration approaches those of MMT and TiO_2_, a drop in ethylene removal efficiency occurs. This trend becomes more evident when analyzing the results of Samples Amt 6 and Amt 8 that despite having the same MMT concentration, both showed reduced performance, possibly attributed to the higher silver concentration compared to Samples Amt 2 and Amt 4. The presence of silver, in some systems, can exert a negative effect, acting to obstruct the pores of the support materials, as discussed by Lenzi et al. and Yu et al., corroborated by the pore volume values observed in the silver-containing samples.^47,62^ An additional interesting aspect is the comparison between Samples Amt 6 and Amt 8. The Amt 6 exhibited lower removal efficiency compared to Amt 8, despite both containing the same amount of MMT. This behavior can be explained by the higher TiO_2_ concentration in Amt 8, which may be associated with a larger pore volume, thus favoring better performance in ethylene removal. This pattern can be largely observed across the samples.

### 3.6 Contour Plots by Statistica Software

Based on the data obtained in the response, the interactions among the experimental factors were analyzed with the aid of Minitab software version 21.4.1, generating the contour plots presented in Figure 5.

**Figure 5:**
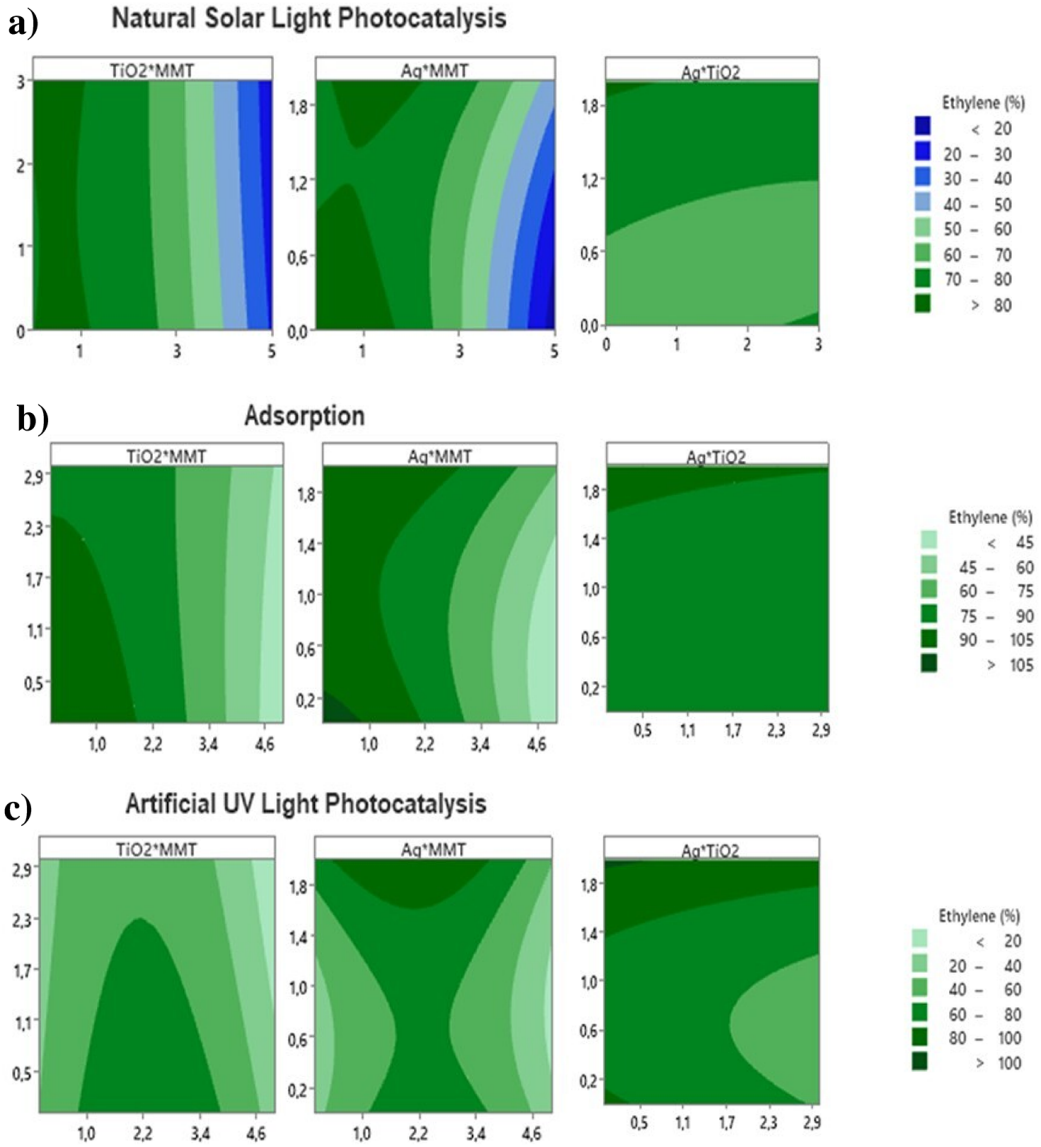
Contour plots for ethylene removal via: photocatalysis under natural (solar) light (a), adsorption (b), and photocatalysis under artificial UV light (c).

In the adsorption scenario, it is observed that the samples exhibited low efficiency in ethylene removal. TiO_2_, both pure and in combination with MMT and Ag components, did not perform well, which was expected, as it is not considered a good adsorbent. The presence of MMT provided a slight improvement, especially in the Ag*MMT system. Nonetheless, the results were limited, highlighting the low efficacy of the isolated adsorption process.

In contrast, in the artificial UV light photocatalysis scenario, the graphs exhibit areas of higher removal efficiency. The TiO_2_*MMT system stood out by showing the lowest residual ethylene concentrations, suggesting an effective synergy between adsorption (promoted by MMT) and photocatalytic degradation (promoted by TiO_2_). The Ag*MMT combination also performed well, although inferior to the TiO_2_*MMT system, and the Ag*TiO_2_ system showed intermediate efficiency. These results confirm that photocatalysis under artificial UV light substantially enhances the removal efficiency, especially when TiO_2_ is associated with a support like MMT.

In the natural (solar) light photocatalysis scenario, areas corresponding to high ethylene removal values and, consequently, excellent performance are verified in the TiO_2_*MMT and Ag*MMT systems. In general, natural (solar) light photocatalysis showed a positive effect in some combinations, possibly due to the partial activation of TiO_2_, even in the absence of intense UV radiation. This may be influenced by the presence of silver in the composition, given that Ag reduces the activation energy, as reported by Abbad et al.^73^ Despite this, the Ag*TiO_2_ system maintained an intermediate efficiency, inferior to the MMT containing systems. These results suggest that the TiO_2_*MMT system can act synergistically, combining adsorption and a possible partial photocatalytic activation, justifying the high performance observed, as documented in works by Kang et al., Li et al. and Zeng et al.^74–76^

Overall, the comparison among the evaluated scenarios highlights the TiO_2_*MMT system as the most efficient, both in artificial and natural light photocatalysis, due to the synergy between adsorption and catalytic degradation. In the case of isolated adsorption, on the other hand, there was no significant distinction among the systems, evidencing the limitation of this process when applied individually.

Based on the results obtained, the Minitab software indicated an optimal formulation point with a concentration of 5% MMT and 2.73% TiO_2_. This composition, designated as Sample 21 (Amt 21), was synthesized, heat-treated, and applied in the same reaction scenarios as the experimental treatment samples, as presented in Figure 6.

**Figure 6:**
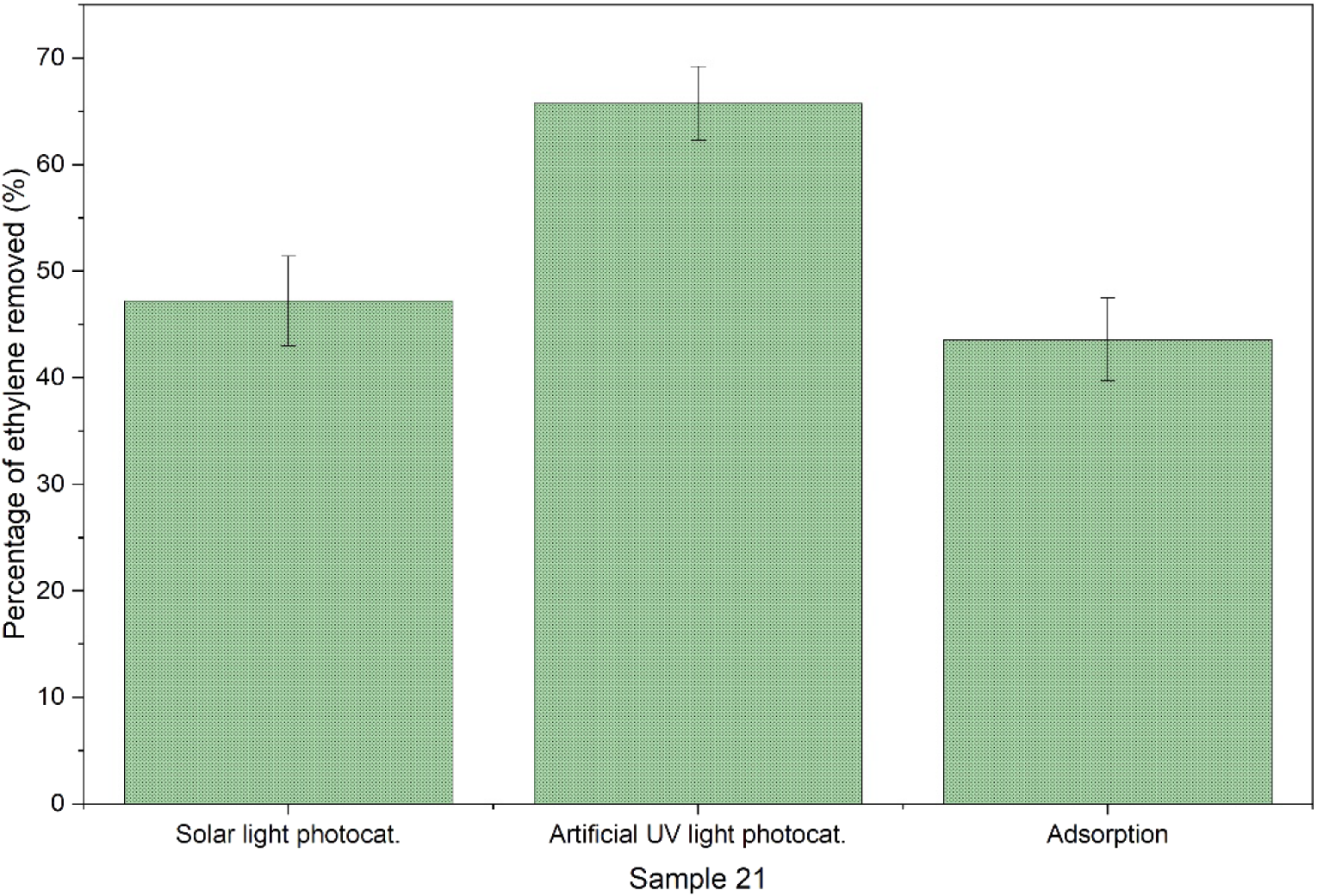
Evaluation of optimal sample AMT 21 (5% MMT and 2.73% TiO_2_) in reaction scenarios.

Figure 6 presents the behavior of Sample 21 in the same scenarios for ethylene removal in mean ± SD for each scenario. It is verified that, in the photocatalysis reaction under artificial UV light, the sample achieved the highest percentage of ethylene removal, with approximately 65%, demonstrating the efficiency of the degradation process. In the natural (solar) light photocatalysis scenario, the sample exhibited satisfactory performance with about 47% removal, values close to that observed in the adsorption scenario with 44% removal from the reaction system.

## 4 Conclusions

This study reports the synthesis and application of MMT/TiO_2_/Ag composites as efficient materials for ethylene removal. Experimental design and performance evaluation across multiple scenarios revealed high removal efficiency. Notably, sample AMT 09 (1.0% Ag and 1.5% TiO2) demonstrated superior photocatalytic activity, reaching an outstanding ethylene removal rate of over 92% within 120 min under artificial UV irradiation. This performance significantly outperformed natural sunlight conditions, which yielded less than 16% degradation, and adsorption tests, which showed nearly no removal capacity. Sample AMT 10 (5% MMT, 1.5% TiO_2_, and 1% Ag) demonstrated a remarkable balance, maintaining high efficiency across all tested conditions: adsorption (62.69%), artificial UV (76.90%), and natural sunlight (74.05%), suggesting a positive synergistic interaction between the composite components. Statistical optimization identified an ideal formulation (Sample AMT 21:5% MMT and 2.73% TiO_2_), which was experimentally validated. This optimized sample achieved significant removal rates of 65% under UV light and 47% under natural sunlight, confirming the robustness of the statistical model. In conclusion, the MMT/TiO_2_/Ag and TiO_2_/Ag system represents a promising, low-cost, and sustainable alternative for postharvest management. Its capability to degrade ethylene under both artificial and natural light sources positions it as a viable technology for integration into food packaging and coatings, potentially extending the shelf life of fresh produce, such as bananas, and contributing to the reduction of global food waste.

## Supplementary Material



## Notes

X-ray diffraction (XRD) patterns of the calcined and uncalcined AMT 10 samples (PDF)
