# Supplementary material for: Design of Experiments for the Synthesis of MMT/TiO2/Ag Composites: Integrating Adsorption and Photocatalysis under Artificial UV and Natural Sunlight for Ethylene Degradation
Source: ACS Omega. 2026 Jun 30;11(27):40030–42. doi: 10.1021/acsomega.6c01808 (PMC13382735; doi:10.1021/acsomega.6c01808)
Supplement: Supplementary file 1 [file ao6c01808_si_001.pdf]

Supporting Information

Design of Experiments for the Synthesis of MMT/TiO_2/Ag Composites: Integrating Adsorption and Photocatalysis under Artificial UV and Natural Sunlight for Ethylene Degradation

Yuri B. Fávaro[1]; Michel Z. Fidelis[2]; Giane G. Lenzi[3]; Artur J. Motheo[2]; Marcos David Ferreira[4]; Henriette M. C. de Azeredo[1,4]*

[1]Graduate Program in Chemical Engineering (PPGEQ), Federal University of São Carlos (UFSCAR), São Carlos, São Paulo 13565-905, Brazil

[2]São Carlos Institute of Chemistry, University of São Paulo (USP), São Carlos, São Paulo 13566-590, Brazil

[3]Department of Chemical Engineering, Federal Technological University of Paraná (UTFPR), Ponta Grossa, Paraná 84017-220, Brazil

[4]Embrapa Instrumentation, São Carlos, São Paulo 13560-970, Brazil

*E-mail: yurifavaro@estudante.ufscar.br

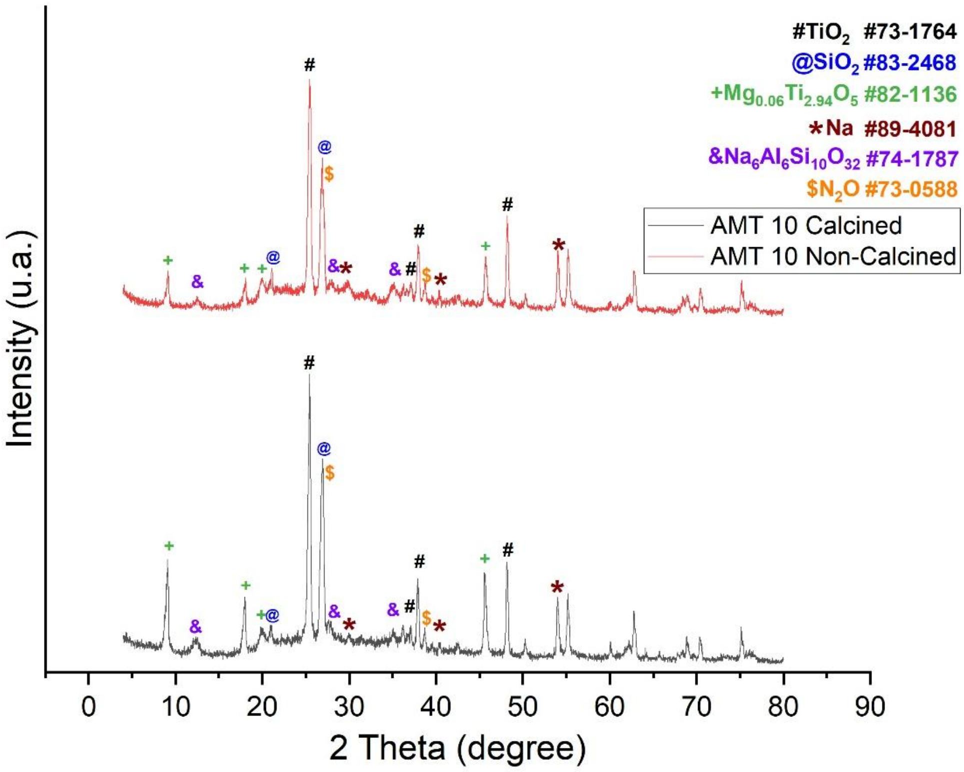

Figure S1. X-ray diffraction (XRD) patterns of the MMT/TiO2/Ag composites for Samples AMT 10 calcined and AMT 10 non-calcined.
